# Increasing the reference populations for the 55 AISNP panel: the need and benefits

**DOI:** 10.1007/s00414-016-1524-z

**Published:** 2017-01-09

**Authors:** Andrew J. Pakstis, Longli Kang, Lijun Liu, Zhiying Zhang, Tianbo Jin, Elena L. Grigorenko, Frank R. Wendt, Bruce Budowle, Sibte Hadi, Mariam Salam Al Qahtani, Niels Morling, Helle Smidt Mogensen, Goncalo E. Themudo, Usha Soundararajan, Haseena Rajeevan, Judith R. Kidd, Kenneth K. Kidd

**Affiliations:** 1 Department of Genetics, Yale University School of Medicine, P.O. Box 208005, 333 Cedar Street, New Haven, CT 06520-8005 USA; 2 Key Laboratory for Molecular Genetic Mechanisms and Intervention Research on High Altitude Disease of Tibet Autonomous Region, School of Medicine, Xizang Minzu University, Xianyang, Shaanxi 712082 China; 3 Key Laboratory of High Altitude Environment and Genes Related to Disease of Tibet Ministry of Education, School of Medicine, Xizang Minzu University, Xianyang, Shaanxi 712082 China; 4 Department of Psychology, University of Houston, Houston, TX 77204 USA; 5 Department of Molecular and Human Genetics, Baylor College of Medicine, Houston, TX 77030 USA; 6 Institute of Molecular Medicine, University of North Texas Health Science Center, 3500 Camp Bowie Blvd, Fort Worth, TX 76107 USA; 7 Center for Human Identification, University of North Texas Health Science Center, 3500 Camp Bowie Blvd, Fort Worth, TX 76107 USA; 8 Center of Excellence in Genomic Medicine Research, King Abdelaziz University, Jeddah, Saudi Arabia; 9 School of Forensic & Applied Sciences, University of Central Lancashire, Preston, UK; 10 Section of Forensic Genetics, Department of Forensic Medicine, University of Copenhagen, DK-2100 Copenhagen, Denmark

**Keywords:** Ancestry, SNP, Reference database, FROG-kb, Alfred

## Abstract

Ancestry inference for an individual can only be as good as the reference populations with allele frequency data on the SNPs being used. If the most relevant ancestral population(s) does not have data available for the SNPs studied, then analyses based on DNA evidence may indicate a quite distantly related population, albeit one among the more closely related of the existing reference populations. We have added reference population allele frequencies for 14 additional population samples (with >1100 individuals studied) to the 125 population samples previously published for the Kidd Lab 55 AISNP panel. Allele frequencies are now publicly available for all 55 SNPs in ALFRED and FROG-kb for a total of 139 population samples. This Kidd Lab panel of 55 ancestry informative SNPs has been incorporated in commercial kits by both ThermoFisher Scientific and Illumina for massively parallel sequencing. Researchers employing those kits will find the enhanced set of reference populations useful.

**Electronic supplementary material:**

The online version of this article (doi:10.1007/s00414-016-1524-z) contains supplementary material, which is available to authorized users.

## Introduction

Soundararajan et al. [1] recently highlighted the limited utility of the many published ancestry informative SNP (AISNP) panels. The review identified 21 publications reporting different SNP panels for ancestry inference. The union of SNPs in the 21 published panels consisted of 1397 SNPs of which only 46 occurred in three to six panels. No SNP occurred in more than six of the 21 panels. Also, relatively few ethnic groups had been studied on any common set of SNPs making comparisons and likelihood calculations difficult to impossible for forensic ancestry applications. The development efforts underlying some panels involved examining population samples from only a few very different world regions. The review concluded that there is little need for more new ancestry panels focused on inferring ancestry to a handful of major world geographical regions. What is needed is a coordination of efforts to greatly expand the ethnic populations with published SNP frequency data on the panels with the best worldwide coverage of human diversity. Soundararajan et al. (2016) [1] note that the 128 AISNPs from the Seldin group [2, 3] and our Kidd Lab set of 55 AISNPs [4, 5] at present have the largest numbers of reference populations with the broadest coverage of major world regions.

Here, we report on 14 additional population samples with allele frequencies on all of the 55 AISNPs in the Kidd lab panel [4]. ALFRED and FROG-kb now have a total of 139 reference populations that have allele frequencies on all 55 of the Kidd panel AISNPs.

## Materials and methods

The 14 new populations are listed in Table 1 with the sample size, the laboratory generating the data, and the typing method employed. Supplementary Table S1 lists the 139 different population samples representing the diverse ethnic groups and biogeographic regions that have now been analyzed for these 55 AISNPs. The populations in the table are organized by geographic region. The table also includes the number of individuals, the three-character abbreviations used in illustrations, and the unique sample identifier (UID) in the ALFRED database for looking up the description of each sample.

**Table 1:** The 14 new reference populations for the 55 AISNP panel

Geographical region and population sample description	Sample size (*N*)	Sample unique identifier: ALFRED database	Data source and typing method footnote
Southwest Asia			
Saudi, Saudi Arabia	91	SA004393T	1
Arabs, Abu Dhabi, United Arab Emirates	69	SA004394U	2
East Asia			
Uygur, Xinjiang, China	100	SA004301I	3
Mongols, Inner Mongolia, China	100	SA004303K	3
Hui, Ningxia, China	100	SA004304L	3
Han—Northwest, Shaanxi, China	100	SA004305M	3
Han—Southwest, Yunnan, China	100	SA004307O	3
Tibetans, Southwest Tibet, China	100	SA004302J	3
Miao = Hmong, Guizhou, China	100	SA004306N	3
Li = Hlai, Hainan, China	100	SA004308P	3
Greenland			
Native Greenlanders, Greenland	104	SA004396W	4
North America			
Yavapai, Arizona, USA	62	SA004395V	5
Plains AmerIndians	56	SA000023F	1
Southwest AmerIndians	51	SA000025H	1

1. Genotypes generated at Kidd Lab used the standard TaqMan assays employed previously for the 55 AISNP panel [4, 5]; Saudi DNA supplied by Elena Grigorenko; samples are from healthy individuals who partially overlap with normal controls in Ercan-Sencicek et al., 2015 [6]

2. Allele frequencies for SNPs contributed by Sibte Hadi and colleagues, University of Central Lancashire. Abu Dhabi sample genotypes were identified using the HID Ancestry panel (ThermoFisher Scientific) on the Ion Torrent PGM

3. Genotypes supplied by Longli Kang and colleagues, Xizang Minzu University. SNP genotyping was done primarily with the Sequenom MassARRAY RS1000 following manufacturer’s standard protocol [7]. For four SNPs, genotyping was based on standard TaqMan assays as described for the 55 AISNP panel [4, 5]

4. Genotypes provided by Niels Morling and colleagues, University of Copenhagen. Typing method described in [8]

5. Genotypes contributed by Frank Wendt, Bruce Budowle, and colleagues, University of North Texas Health Science Center. See [9] for typing method details

Every locus-population combination for which individual genotypes were available was tested for Hardy-Weinberg on the assumption that each locus was a codominant di-allelic genetic system. Genotypes were examined to ensure that the alleles on the positive strand have been entered into the database and are used in FROG-kb.

The STRUCTURE [10] software provides one way of assessing how well a set of loci tested on multiple individuals can infer ancestry. We employed version 2.3.4 applying the standard admixture model assuming correlated allele frequencies. At each *K* value from 6 to 10, the program was run 20 times with 10,000 burn-ins and 10,000 Markov Chain Monte Carlo (MCMC) iterations.

## Results

No significant deviations from Hardy-Weinberg ratios were observed beyond those expected by chance. All of the different typing methods appeared consistent: the same alleles are being detected at the same loci. Allele frequencies for the complete set of 55 SNPs in all 139 population samples are accessible in ALFRED. Additional populations have been studied and reported in scientific publications for some of the SNPs, and thus, some SNPs have frequency data on more than 139 population samples in ALFRED. In FROG-kb, the “Kidd Lab—Set of 55 AISNPs” has complete allele frequency data on all 55 SNPs for all 139 reference population samples. The completeness of the data allows likelihoods and likelihood ratios to be calculated for all of these 139 population samples for any input DNA profile for the 55 AISNPs (or a subset of the SNPs).

**Fig. 1:**
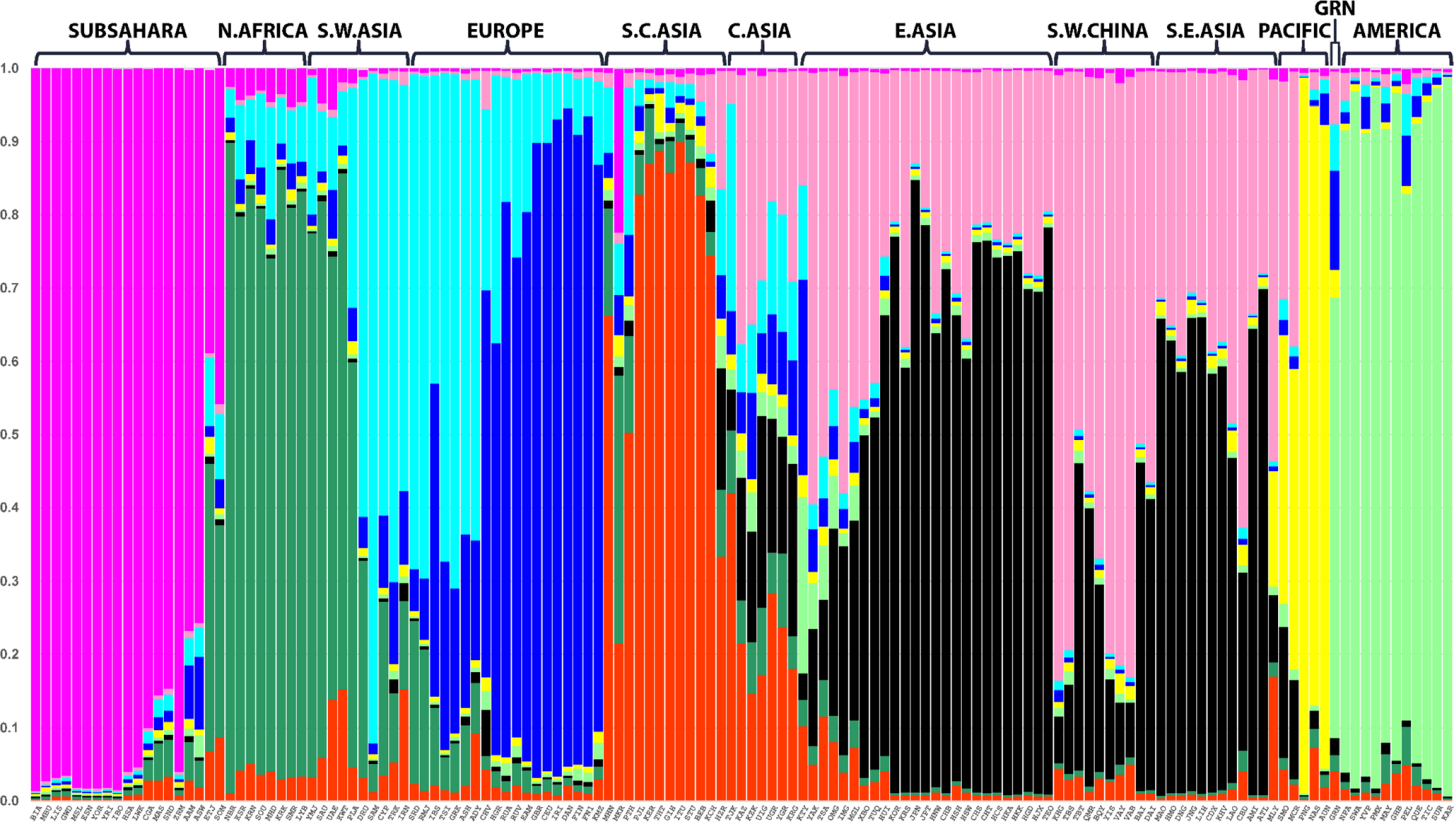
STRUCTURE results for estimated cluster membership values at *K* = 9 in 139 reference populations. Population abbreviations are explained in Table S1

The STRUCTURE analysis result displayed in Fig. 1 is for the highest likelihood run of the most commonly occurring (for 10 of 20 runs) cluster pattern at *K* = 9. This analysis includes 66 additional populations since the previously reported analysis of only 73 populations [4]. The analysis of 139 reference populations included 8055 individuals after excluding individuals with an excessive number of missing genotypes. Of the 8055 individuals analyzed, 72.3% had all 55 AISNP genotypes present; 91.0% of individuals had no more than three missing typings; 1.9% of the 55 × 8055 possible genotypes were missing. The new populations generally show strong similarity to previously analyzed populations that were known to be closely related. In addition, a group of related populations from a previously underrepresented region can clearly define a new cluster, witness the North African cluster. Another interesting new clinal pattern across clusters for East Asian populations is seen for the ethnic minority populations sampled in Southwestern China—primarily Tibet, Yunnan, and Sichuan provinces. Elsewhere, a group of populations from Central Asia appears similar but shows partial similarity to several of the inferred clusters that predominate among populations in the surrounding geographical regions.

## Discussion and conclusions

With nearly twice as many population samples, we can see significant differences when that previously published STRUCTURE result [4] is compared with this new one. The 55 AISNP panel now shows that an additional cluster is defined by the North African populations recently included [5], consistent with the findings based on many more SNPs, that these North African populations form a genetically distinct cluster [11]. The large number of additional East Asian populations begins to show variation among the populations with Tibetans and other ethnic groups from Southwest China having a pattern of ancestral similarity that is quite visually distinct. Strong frequency differences at five SNPs (near ADH1B, ALDH2, OCA2, RPS28P8) underlie the Southwest China pattern obtained from STRUCTURE. The Southwest China pattern we have observed may correspond to the distinctive ethnic patterns reported for a study of mitochondrial DNA in 115 populations in Southern Asia [12]. They reported an interesting clustering of distinctive haplotypes centered on Myanmar and Southwest China.

Our interpretation of the multiple cluster memberships for populations in Central Asia is that this set of AISNPs does not work well to distinguish Central Asian populations from populations to the East, South, and West. While, historically, Central Asia has seen many migrations, it has been inhabited since modern humans first reached this far in their expansion from Africa before they reached East Asia. How much the historical migrations are responsible compared to these simply being intermediate and not well differentiated by these SNPs is a question for future research.

In our update on the growing number of reference populations studied for the 55 AISNPs [5], our previous conclusion remains very relevant; specifically, “The ideal forensic ancestry inference resource will consist of a large number of highly informative AISNPs with full data on a large number of population samples representing all regions of the world.” Finding the best and most appropriate ancestry match for an individual in forensic work depends on having a comprehensive set of reference populations from around the world. Many more reference populations are needed. As various relatively neglected geographical regions and smaller ethnic groups in better studied areas are added, we will likely observe more interesting new cluster patterns and novel clinal variations. Such new findings will offer fresh opportunities to improve and fine-tune the best AISNP panels that will develop.

We continue to work on our own and with our collaborators to study more new populations on the 55 AISNP panel. We also continue to assess other SNPs for ancestry informativeness and whether they can improve refined ancestry inference by modifying the existing panel. We continue to encourage other researchers to consider adding their unique populations to this growing dataset of population samples which are all tested for the same set of ancestry informative SNPs. Similarly, we encourage others with excellent candidate AISNPs to request that we test them on our population samples.

These results demonstrate the value of more populations studied for a small number of informative SNPs. To date, no other panel of AISNPs has data on such a large number of populations distributed as widely. That does not mean this panel should be considered a final panel. There are other panels, noted above, that are also included in ALFRED and FROG-kb. We are working to increase reference population coverage of those panels as well so that the most globally informative subset of SNPs can be identified from the union.

## Electronic supplementary material

Supplementary Table S1 The 139 population samples in STRUCTURE analyses ordered in bar graph Fig. 1. Three-character population abbreviations are explained in Table S1. (PDF 88.9 kb)
