# Supplementary material for: Increasing the reference populations for the 55 AISNP panel: the need and benefits
Source: Int J Legal Med. 2017 Jan 9;131(4):913–7. doi: 10.1007/s00414-016-1524-z (PMC5491587; doi:10.1007/s00414-016-1524-z)
Supplement: Supplementary file 1 — The 139 population samples in STRUCTURE analyses ordered in bar graph Fig. 1. Three-character population abbreviations are explained in Table S1. (PDF 88.9 kb) [file 414_2016_1524_MOESM1_ESM.pdf]

Supplementary Material

Submitted to *International Journal of Legal Medicine* for paper titled:

"Increasing the reference populations for the 55 AISNP panel: the need and benefits"

Andrew J. Pakstis [a], Longli Kang[b], Lijun Liu[b], Zhiying Zhang[b], Tianbo Jin[b], Elena Grigorenko[c], Frank R.Wendt[d], Bruce Budowle[e], Sibte Hadi[f], Mariam Salam Al Qahtani[f,] Niels Morling[g], Helle Smidt Mogensen[g], Goncalo E. Themudo[g], Usha Soundararajan[a], Haseena Rajeevan[a], Judith R. Kidd[a], Kenneth K. Kidd[a]

[a] Department of Genetics, Yale University School of Medicine, New Haven, CT 06520 USA

[b] Key Laboratory for Molecular Genetic Mechanisms and Intervention Research on High Altitude Disease of Tibet Autonomous Region *and* Key Laboratory of High Altitude Environment and Genes Related to Disease of Tibet Ministry of Education, School of Medicine, Xizang Minzu University, Xianyang, Shaanxi 712082, China

[c] Developmental Cognitive Neuroscience, University of Houston, Houston, TX 77204 USA

[d] Institute of Molecular Medicine, University of North Texas Health Science Center, 3500 Camp Bowie Blvd. Fort Worth, TX 76107, USA

[e] Center for Human Identification, University of North Texas Health Science Center, 3500 Camp Bowie Blvd, Fort Worth, Texas 76107 USA; Center of Excellence in Genomic Medicine Research, King Abdelaziz University, Jeddah, Saudi Arabia

[f] School of Forensic & Applied Sciences, University of Central Lancashire, Preston, UK

[g] Section of Forensic Genetics, Department of Forensic Medicine, University of Copenhagen, DK-2100, Copenhagen, Denmark

---

Table S1. The 139 population samples in STRUCTURE analyses ordered as in bar graph Figure 1.

N [a] Indiv.	World Region	Abbrev.	Population Name [b]	Data Source For Genotypes [c]
69	SubSahara	BIA	Biaka	Kidd Lab
39		MBU	Mbuti	Kidd Lab
8		LIS	Lisongo	Kidd Lab
113		GWD	Gambians	1KG
85		MSL	Mende	1KG
99		ESN	Esan	1KG
77		YOR	Yoruba	Kidd Lab
108		YRI	Yoruba	1KG
48		IBO	Ibo	Kidd Lab
39		HSA	Hausa	Kidd Lab
99		LWK	Luhya	1KG
45		CGA	Chagga	Kidd Lab
20		MAS	Masai	Kidd Lab
40		SND	Sandawe	Kidd Lab
40		ZRM	Zaramo	Kidd Lab
89		AAM	African Americans	Kidd Lab
61		ASW	African Americans	1KG

45		ETJ	Ethiopian Jews	Kidd Lab
20		SOM	Somali	Kidd Lab
11	N Africa	NBR	Nebeur	Kidd Lab
43		KSR	Kesra	Kidd Lab
46		KRN	Kairoun	Kidd Lab
47		SOU	Sousse	Kidd Lab
40		MHD	Mehdia	Kidd Lab
46		KRK	Kerkennah	Kidd Lab
62		SMR	Smar	Kidd Lab
70		LYB	Libyans	Kidd Lab
42	SW Asia	YMJ	Yemenite Jews	Kidd Lab
91		SAU	Saudi	Kidd Lab
38		UAE	Arabs, Abu Dhabi, UAE	S. Hadi
14		KWT	Kuwaiti	Kidd Lab
69		PLA	Palestinians	Kidd Lab
102		DRU	Druze	Kidd Lab
39		SAM	Samaritans	Kidd Lab
59		CYP	Turkish Cypriots	Kidd Lab
80		TRK	Turkish	Kidd Lab
44		IRN	Iranians	Kidd Lab
35	S Europe	SRD	Sardinians	Kidd Lab
27		RMJ	Roman Jews	Kidd Lab
107		IBS	Iberians	1KG
107		TSI	Toscani	1KG
55		GRK	Greeks	Kidd Lab
140		ASH	Ashkenazi	Kidd Lab
54		ADY	Adygei	Kidd Lab
42	N Europe	CHV	Chuvash	Kidd Lab
89		HGR	Hungarians	Kidd Lab
33		RUA	Russians, Archangelsk	Kidd Lab
47		RUV	Russians, Vologda	Kidd Lab
89		EAM	European Americans	Kidd Lab
91		GBR	British	1KG
99		CEU	CEPH Europeans	1KG
114		IRI	Irish	Kidd Lab
51		DAN	Danes	Kidd Lab
34		FIN	Finns	Kidd Lab
99		FN1	Finns	1KG
47	W Siberia	KMZ	Komi Zyriane	Kidd Lab
52	SC Asia	MHN	Mohanna	Kidd Lab
26		MKR	Makrani	Kidd Lab
122		PTH	Pathans	Kidd Lab
96		PJL	Punjabi	1KG
30		KER	Keralites	Kidd Lab
14		THT	Thoti	Kidd Lab
103		GIH	Gujarati	1KG
102		ITU	Telugu	1KG
102		STU	Tamil (Sri Lanka)	1KG
86		BEB	Bengali	1KG
17		KCH	Kachari	Kidd Lab
104		HZR	Hazara	Kidd Lab
20	C Asia	TJK	Tajik	C.-X. Li
45		KAZ	Kazakhs	H. Li

30		KZK	Kazakhs	C.-X. Li
46		UIG	Uyghur	H. Li
30		UGR	Uyghur	C.-X. Li
100		YGR	Uyghur	L. Kang
30		KRG	Kirghiz,	C.-X. Li
50	E Siberia	KTY	Khanty	Kidd Lab
51		YAK	Yakut	Kidd Lab
51	E Asia	TSA	Tsaatan	Kidd Lab
71		OMG	Outer Mongolians	Kidd Lab
68		IMG	Inner Mongolians	H. Li
100		MGL	Inner Mongolians	L. Kang
29		XBO	Xibo, Xinjiang	C.-X. Li
26		TUQ	Tu, Qinghai	C.-X. Li
100		HUI	Hui, Ningxia	L. Kang
54		KOR	Koreans	Kidd Lab
34		KRS	Koreans	C.-X. Li
48		JPN	Japanese	Kidd Lab
104		JPT	Japanese	1KG
100		HNW	Han, Northwest	L. Kang
103		CHB	Han, Beijing	1KG
47		HSH	Han, Shandung	C.-X. Li
100		HSW	Han, Southwest	L. Kang
58		CHS	Chinese, “Cantonese”	Kidd Lab
50		CHT	Han, Taiwan	Kidd Lab
105		HCS	Southern Han	1KG
41		HKA	Hakka	Kidd Lab
50		HKK	Hakka	C.-X. Li
40		HGD	Han, Guangdong	C.-X. Li
28		HJX	Han, Guangxi	C.-X. Li
56		TEO	Han, Teochew speakers	C.-X. Li
30	SW China	KHG	Khamba Tibetans, Sichuan	H. Li
100		TBS	Tibetans	C.-X. Li
50		TBT	Tibetans	L. Kang
39		QMR	Qiang, Sichuan	H. Li
42		BQY	Baima Dee, Sichuan	H. Li
26		YIS	Yi, Sichuan	C.-X. Li
29		VAY	Va, Yunnan	C.-X. Li
16		VAB	Va, Myanmar	C.-X. Li
30		DAI	Dai, Yunnan	C.-X. Li
23		BAI	Bai, Yunnan	C.-X. Li
25	SE Asia	MAO	Miao=Hmong, Guangxi	C.-X. Li
100		HMO	Miao=Hmong, Guizhou	L. Kang
28		DNG	Dong, Guangxi	C.-X. Li
27		JNG	Jing, Gunagxi	C.-X. Li
100		LIH	Li = Hlai, Hainan	L. Kang
93		CDX	Dai	1KG
99		KHV	Vietnamese	1KG
118		LAO	Laotians	Kidd Lab
24		CBD	Cambodians	Kidd Lab
40		AMI	Ami, Taiwan	Kidd Lab
42		ATL	Atayal, Taiwan	Kidd Lab
11		MLY	Malaysians	Kidd Lab
9	Pacific	SMO	Samoans	Kidd Lab

34		MCR	Micronesians	Kidd Lab
22		PNG	Papuans, New Guinea	Kidd Lab
23		NAS	Nasioi	Kidd Lab
51		AUN	Australian Aborigines	Kidd Lab
104	Greenland	GRN	Greenlanders	N. Morling
56	Americas	NPA	Plains AmerIndians	Kidd Lab
51		SWA	Southwest AmerIndians	Kidd Lab
62		YVP	Yavapai	F. Wendt, B. Budowle
53		PMX	Pima, Mexico	Kidd Lab
50		MAY	Maya, Yucatan	Kidd Lab
12		GHB	Guihiba	Kidd Lab
85		PEL	Peruvians	1KG
22		QUE	Quechua	Kidd Lab
65		TIC	Ticuna	Kidd Lab
43		SUR	Rondonian Surui	Kidd Lab
55		KAR	Karitiana	Kidd Lab

Table S1 footnotes:

[a] Sample sizes here correspond to the number of individuals included in STRUCTURE analyses.

[b] See Table 1 footnotes for more details on sample collection and genotyping for the 15 newest reference population samples. See footnotes in Table S1 of [5] Pakstis et al. (2015) for information concerning sources for collection of previously contributed reference population samples.

[c] 1KG abbreviation stands for the 1000 Genomes Project--Phase 3.
